# The EMBL-EBI Job Dispatcher sequence analysis tools framework in 2024

**DOI:** 10.1093/nar/gkae241

**Published:** 2024-04-10

**Authors:** Fábio Madeira, Nandana Madhusoodanan, Joonheung Lee, Alberto Eusebi, Ania Niewielska, Adrian R N Tivey, Rodrigo Lopez, Sarah Butcher

**Affiliations:** European Molecular Biology Laboratory, European Bioinformatics Institute (EMBL-EBI), Wellcome Trust Genome Campus, Hinxton, Cambridge CB10 1SD, UK; European Molecular Biology Laboratory, European Bioinformatics Institute (EMBL-EBI), Wellcome Trust Genome Campus, Hinxton, Cambridge CB10 1SD, UK; European Molecular Biology Laboratory, European Bioinformatics Institute (EMBL-EBI), Wellcome Trust Genome Campus, Hinxton, Cambridge CB10 1SD, UK; European Molecular Biology Laboratory, European Bioinformatics Institute (EMBL-EBI), Wellcome Trust Genome Campus, Hinxton, Cambridge CB10 1SD, UK; European Molecular Biology Laboratory, European Bioinformatics Institute (EMBL-EBI), Wellcome Trust Genome Campus, Hinxton, Cambridge CB10 1SD, UK; European Molecular Biology Laboratory, European Bioinformatics Institute (EMBL-EBI), Wellcome Trust Genome Campus, Hinxton, Cambridge CB10 1SD, UK; European Molecular Biology Laboratory, European Bioinformatics Institute (EMBL-EBI), Wellcome Trust Genome Campus, Hinxton, Cambridge CB10 1SD, UK; European Molecular Biology Laboratory, European Bioinformatics Institute (EMBL-EBI), Wellcome Trust Genome Campus, Hinxton, Cambridge CB10 1SD, UK

## Abstract

The EMBL-EBI Job Dispatcher sequence analysis tools framework (https://www.ebi.ac.uk/jdispatcher) enables the scientific community to perform a diverse range of sequence analyses using popular bioinformatics applications. Free access to the tools and required sequence datasets is provided through user-friendly web applications, as well as via RESTful and SOAP-based APIs. These are integrated into popular EMBL-EBI resources such as UniProt, InterPro, ENA and Ensembl Genomes. This paper overviews recent improvements to Job Dispatcher, including its brand new website and documentation, enhanced visualisations, improved job management, and a rising trend of user reliance on the service from low- and middle-income regions.

## Graphical Abstract

**Graphical Abstract:**
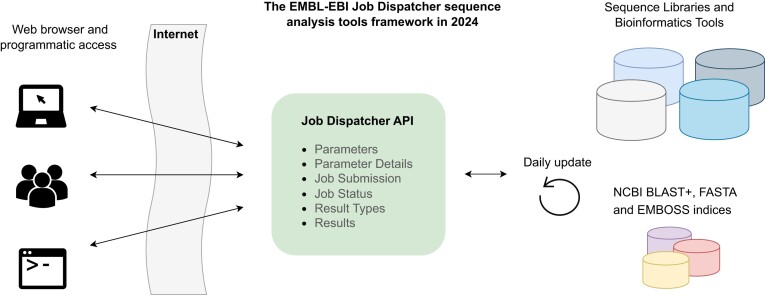

## Introduction

The European Bioinformatics Institute (EMBL-EBI) is one of the world's leading sources of public biomolecular data (1). A wealth of deposition databases, experimental data archives, and added-value knowledge bases that provide annotation, curation, reanalysis, and integration of deposited data are provided by EMBL-EBI resources and portals. Data are made freely available for use by the scientific communities and serve as foundations for countless scientific studies, research programmes, external resources and applications. EMBL-EBI also provides access to software systems that can be downloaded and installed locally, as well as popular ‘on-demand’ bioinformatics services. Examples of such services include EBI Search (2), which provides a free text search and powerful cross-referencing engine powered by EMBL-EBI datasets, and the Job Dispatcher (JD) framework (2). JD is powered by the EMBL-EBI high-performance computing (HPC) infrastructure and provides integrated access to a comprehensive catalogue of bioinformatics applications and related dataset indices. The catalogue of tools includes some of the most popular powerhouses in bioinformatics, from pairwise- and multiple sequence alignment (MSA) tools, such as Clustal Omega (3), Kalign (4) and Mafft (5), sequence similarity search (SSS) applications, such as NCBI BLAST+ (6) and FASTA (7), tools for functional prediction and annotation such as InterProScan 5 (8) and HMMER 3 (9), RNA analysis tools such as R2DT (10), to sequence analysis utilities from the EMBOSS suite (11). JD applications can be freely accessed via webpage interfaces but also through OpenAPI-compliant Application Programming Interfaces (APIs). These APIs are integrated into popular EMBL-EBI resources such as UniProt (12), InterPro (13), ENA (14) and Ensembl Genomes (15). Sequence similarity search tools enable access to sequence indices covering reference proteomes and genomes, all the way to specialised datasets from major database resources hosted at EMBL-EBI. In this paper, we overview the recent improvements and updates made to the Job Dispatcher framework, highlighting JD’s brand new website and documentation and the increasing relevance of the service for users from low- and middle-income countries.

## New website

A brand new Job Dispatcher website is available at https://www.ebi.ac.uk/jdispatcher. The redeveloped website reorganises the tool and documentation pages and adds new features with an emphasis on enriching the experience for both new and advanced users. It utilises a contemporary frontend framework, following responsive design and high accessibility practices. It has been created as a separate frontend application integrating with the backend via JD’s REST API, as opposed to the previous model in which pages were generated server-side in the monolithic application. The new design allows for greater flexibility in the future development of both the frontend and the backend.

### Landing page

**Figure 1.**
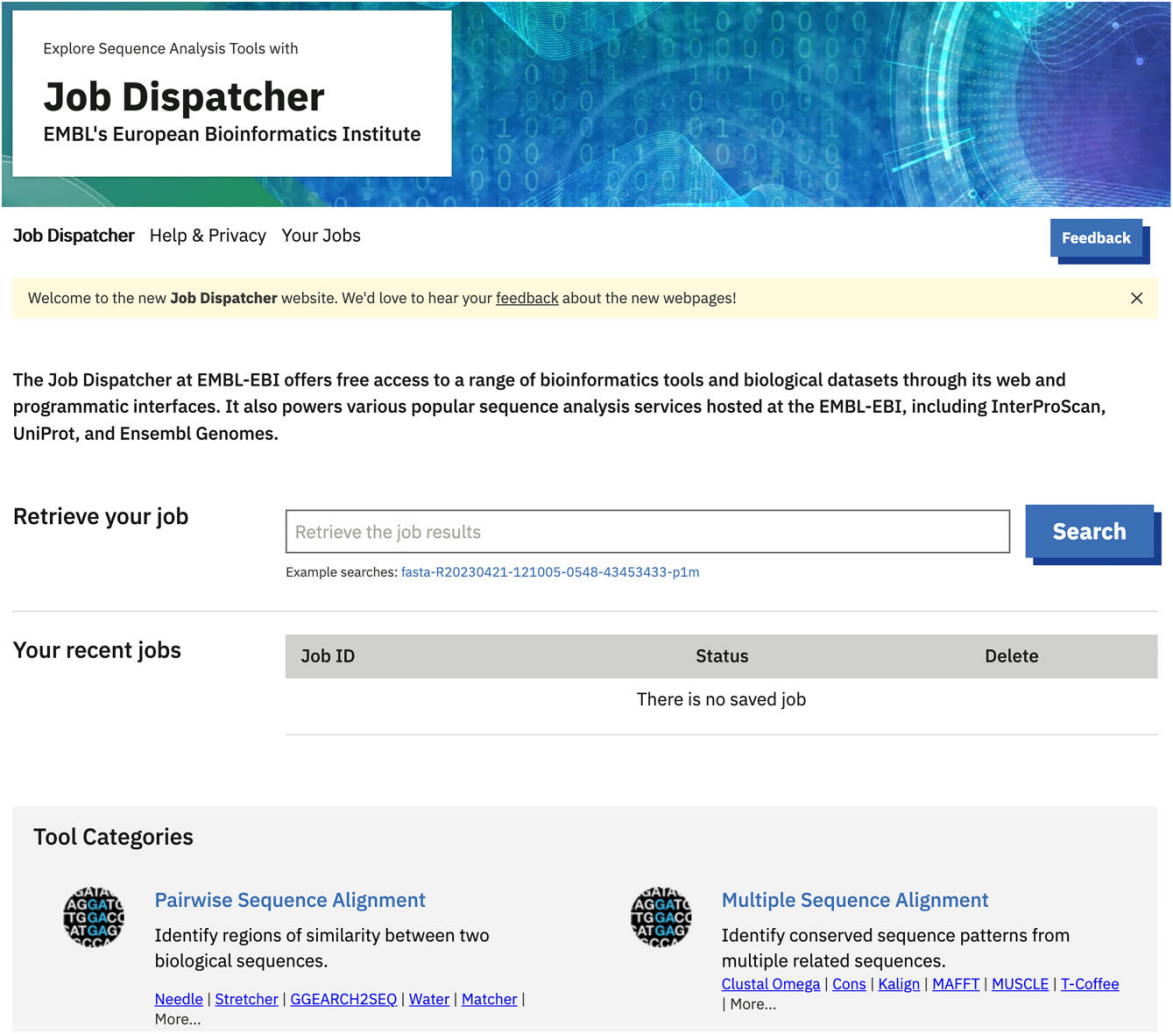
The Job Dispatcher website landing page is available at https://www.ebi.ac.uk/jdispatcher.

The newly introduced landing page (see Figure 1), something that the previous version of the system was missing, offers a user-friendly user interface (UI) that simplifies the navigation of tools across tool categories. This page acts as a one-stop-shop page to access JD bioinformatics tools and results. In addition to expandable tool categories, a job retrieval by Job ID search field and a new recent jobs history view, allows users to search and quickly find their job results. The landing page displays the five most recent jobs allowing users to get instant access to their latest analysis. This page provides sections with relevant service updates and news, a list of JD collaborators and how to cite the service.

### Your jobs

A new ‘Your Jobs’ page provides a history of jobs recently launched by the user. All job IDs are listed for seven days, which matches how long the results are retained for retrieval from the server. It also gives improved information about the job status, which now distinguishes jobs in the HPC ‘queued’ and subsequent ‘running’ states, as well as ‘completed’ and ‘failed’. To improve the findability of individual job IDs, these can be filtered by tool name. Individual or even ‘all’ job IDs can be also removed from the list.

### Tool webforms

To help users identify the most appropriate tool for their analysis, detailed tool descriptions are provided for each linked tool in tool category pages. The tool webforms UI were redesigned to improve the user experience (UX). Relevant information about tool parameters is now provided directly in the form via information popovers, which are displayed on mouse hovering. This information was previously provided via hyperlinks to external documentation pages, therefore simplifying page navigation and improving overall UX. Input validation is performed before job submission. An error message is provided in the webform if the required inputs are not provided or any parameter values are invalid.

### Result pages

The result pages ‘look and feel’ were streamlined across the tools. Several improvements were introduced to all SSS result pages, including a new interactive summary table. The SSS hits can be easily selected and unselected. This enables, for example, downloading the chosen hit sequences in *fasta* format or launching an MSA tool such as Clustal Omega, using them as input directly from the summary table. Other features include showing or hiding sequence annotations and the hit-query pairwise sequence alignments. The table provides pagination by default, which improves the display of very long tables in the browser. ‘Organisms’ facets are also provided, allowing for quickly filtering the sequence hits by originating species. Table sorting, EBI Search (2) cross-references and hyperlinks to the relevant sequence resources are also provided.

### Interactive visualisations

**Figure 2.**
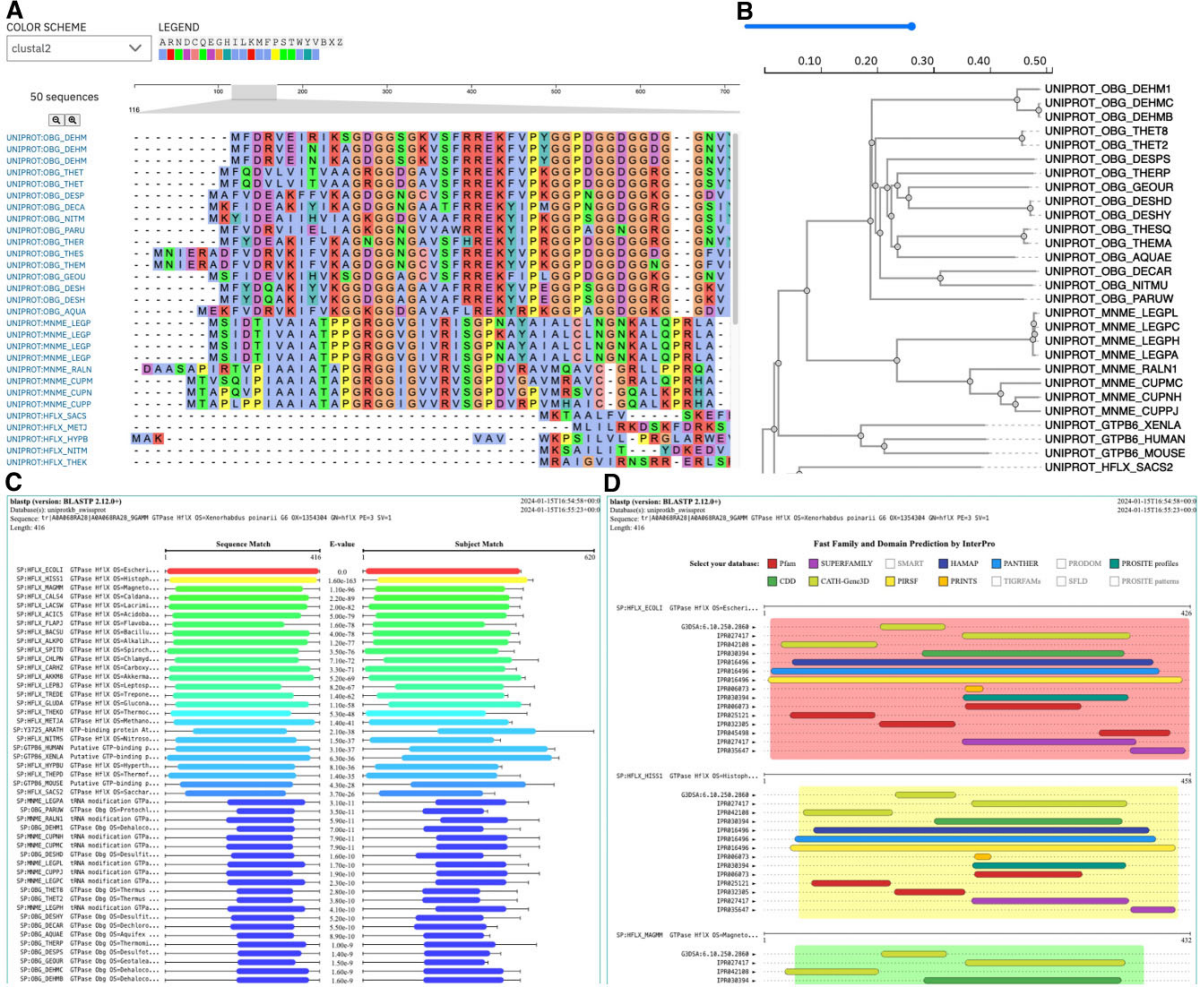
Example interactive visualisations available from JD result pages: (**A**) MSA viewer; (**B**) phylogenetic tree viewer; (**C**) SSS visual output; (**D**) SSS functional predictions by InterPro (13).

Several interactive visualisations are now available throughout the result pages of MSA and SSS tools. The Nightingale (16) MSA viewer is provided as the default view for MSA result pages (see Figure 2A). The MSA viewer provides zoom and navigation controls. It also provides alternative colouring scheme options and the corresponding colour legend. Interactive phylogenetic tree and dendrogram visualisations powered by phylotree.js (17) are also available (see Figure 2B). In addition to general zoom, the visualisation provides several interactive functions, including the selection of terminal, internal and incident branches, in addition to tree branch collapsing and re-rooting. New interactive visualisations are provided for SSS results. Interactive graphical representations of SSS tool outputs and functional predictions provided by InterPro (13) have been developed by us (https://github.com/ebi-jdispatcher/jdispatcher-viewers—see Figure 2C and D, respectively). These can be visualised by clicking on the ‘Visual Output’ and ‘Functional Predictions’ tabs of the SSS result pages, respectively, and provide toggleable colouring and prediction tracks.

### Help & Privacy

The ‘Help & Privacy’ page provides general help and information about JD. A quick overview of how to use the sequence analysis tools is provided. A list of previously delivered webinars and online tutorials can be browsed and accessed through the EMBL-EBI online training (available from https://www.ebi.ac.uk/training/playlists/shared/job-dispatcher-services). Links to our full documentation and FAQs, as well as to the JD blog, are provided. Importantly, links to JD’s Terms of Use and Privacy Notice and contact information are also provided on this page.

## New documentation

The new JD documentation available from https://www.ebi.ac.uk/jdispatcher/docs provides a gateway to key information on a range of topics. Similarly to the ‘Help & Privacy’ page, various links to training materials and outreach activities, news and updates about the service, previous publications, funding information, JD collaborators, and how to contact the team are provided. The documentation expands on two main themes: (i) using the web pages and (ii) programmatic access. The first section provides general information about how to use the tool webforms and how to view the tool results. Examples of common tool inputs and outputs in a variety of file formats, as well as a list of available sequence databases, are provided. The second section provides a general overview of how to use the JD APIs. It provides important notices about the service fair-use policy and describes various resources that are made available to users. For example, OpenAPI specifications, sample clients and CWL (https://commonwl.org/) example workflows. Lastly, an FAQs section is provided, covering the most common user queries. This section covers many issues related to tool output, colour schemes, phylogenetic trees, and other areas.

## Updates on tools and data resources

Sequence analysis tools running under JD are categorised according to their functionality and have been regularly updated to their latest available versions. A list of the bioinformatics tools currently provided by JD and their categories is provided in Supplementary Table S1. As part of our tool consolidation effort, several tools were updated to run in containers with Singularity. To improve findability, JD tools listed in bio.tools (18) have been tagged and provided as the ‘Job Dispatcher Tools’ collection (available from https://bio.tools/t?collectionID=‘JobDispatcherTools’).

Sequence dataset updates and releases are routinely deployed for datasets such as UniProt, PDBe (19), Ensembl Genomes, WormBase Parasite (20), and ENA. To enhance the robustness and efficiency of indexing biological databases required for tools such as FASTA and NCBI BLAST+, the JD data indexing pipelines were migrated to Nextflow (21). UniVec (https://ftp.ncbi.nih.gov/pub/UniVec/) has been added as a new vector contamination sequence dataset for SSS tools. A list of all the datasets currently available within JD is provided in Supplementary Table S2.

## Usage of the services

The JD service has a global reach and plays a vital role in advancing scientific research. In 2023, >109 million JD jobs were performed on EMBL-EBI’s high-performance computing clusters. This corresponds to ∼28 million additional jobs, when compared to 2022, and corresponds to ∼9 million jobs per month and ∼2 million per week. The majority of these jobs (∼94%), were launched programmatically, with the remaining ∼6% submitted through interactive web interfaces. Nevertheless, the largest portion of unique users (∼73%) submitted jobs via the JD frontend. Notably, of the 977 thousand unique users that ran JD jobs in 2023; an increase of 110 thousand from 2022; 61% were from low- and middle-income countries. This represents a 19% increase, from 2022 and suggests an increasing relevance of JD among users from these countries.

## Discussion

JD facilitates the worldwide Life Sciences research community by enriching EMBL-EBI’s data resources and by providing free access to related bioinformatics applications with reliable programmatic and web interfaces. While the demand for EMBL-EBI’s resources and services spiked during the COVID-19 pandemic, use has remained higher than pre-pandemic levels (1). The recent economic uncertainty, with rising energy costs and overall inflation, has posed an enormous challenge to data resources and services, particularly those like JD, which are compute and storage-intensive. Over the last few years, this may have contributed to the growth in worldwide JD use figures, particularly among low-income regions.

Considerable development has been performed to improve the deployment of the new frontend application. This ongoing initiative will be continued, extending towards the backend application. These developments enable the next iteration of the EMBL-EBI Job Dispatcher tools framework. Our goal is to expand the offering of both tools and datasets while maintaining the security, scalability and reliability of the service amid rising computational demands and economic pressures. Demand for bioinformatics training has also continued to increase over the last years and it is our commitment to continue our acknowledged user-support and training initiatives.

## Supplementary Material

gkae241_Supplemental_File

## Data Availability

Job Dispatcher tools are available from https://www.ebi.ac.uk/jdispatcher and https://www.ebi.ac.uk/services. Detailed documentation about how to use the services programmatically is provided at https://www.ebi.ac.uk/jdispatcher/docs. Additionally, users can explore the JD APIs interactively at: https://www.ebi.ac.uk/jdispatcher/docs/webservices/#openapi. Sample clients in Python, Perl and Java, as well as CWL command-line tool definitions and example workflows, are provided on the following GitHub repositories: https://github.com/ebi-jdispatcher/webservice-clients (https://doi.org/10.5281/zenodo.10844991) and https://github.com/ebi-jdispatcher/webservice-cwl (https://doi.org/10.5281/zenodo.10844999), respectively. These services are developed following FAIR principles.
