# Supplementary material for: The EMBL-EBI Job Dispatcher sequence analysis tools framework in 2024
Source: Nucleic Acids Res. 2024 Apr 10;52(W1):W521–5. doi: 10.1093/nar/gkae241 (PMC11223882; doi:10.1093/nar/gkae241)
Supplement: gkae241_Supplemental_File [file gkae241_supplemental_file.pdf]

Supplementary Table 1 - Bioinformatics applications available through JD in 2024.

**Category**	**Tools**
Multiple Sequence Alignment (https://www.ebi.ac.uk/jdispatcher/msa/)	Clustal Omega, Kalign, MAFFT, MUSCLE, T-Coffee, MView, WebPrank
Pairwise Sequence Alignment (https://www.ebi.ac.uk/jdispatcher/psa/)	Needle, Stretcher, Water, Matcher, LALIGN, GeneWise, GGSEARCH2SEQ, SSEARCH2SEQ
Phylogeny Analysis (https://www.ebi.ac.uk/jdispatcher/phylogeny/)	Simple Phylogeny (ClustalW2)
Protein Functional Analysis (https://www.ebi.ac.uk/jdispatcher/pfa/)	InterProScan 5, PfamScan, Phobius, Pratt, RADAR, HMMER3 phmmer, HMMER3 hmmscan
RNA Analysis (https://www.ebi.ac.uk/jdispatcher/rna/)	Infernal cmscan, MapMi, R2DT
Sequence Similarity Search (https://www.ebi.ac.uk/jdispatcher/sss/)	NCBI BLAST+, PSI-BLAST, FASTA, SSEARCH, FASTM/S/F, GGSEARCH, GLSEARCH, PSI-Search, PSI-Search2
Sequence Statistics (https://www.ebi.ac.uk/jdispatcher/seqstats/)	SAPS, Pepinfo, Pepstats, Pepwindow, Cpgplot, Newcpgreport, Isochore, Dotmatcher, Dottup, Dotpath, Polydot
Sequence Translation (https://www.ebi.ac.uk/jdispatcher/st/)	Transeq, Sixpack, Backtranseq, Backtranambig
Sequence Format Conversion (https://www.ebi.ac.uk/jdispatcher/sfc/)	Seqret, MView
Sequence Operation (https://www.ebi.ac.uk/jdispatcher/so/)	Seqcksum
EMBOSS Suite (https://www.ebi.ac.uk/jdispatcher/emboss/)	Needle, Stretcher, Water, Matcher, Transeq, Sixpack, Backtranseq, Backtranambig, Pepinfo, Pepstats, Pepwindow, Cpgplot, Newcpgreport, Isochore, Dotmatcher, Dottup, Dotpath, Polydot, Seqret
Database Entry Fetch (https://www.ebi.ac.uk/Tools/dbfetch/)	Dbfetch (fetching data from 57 domains, see Supplementary Table 2)

Supplementary Table 2 - Sequence libraries available through JD in 2024.

**Category**	**Data**
UniProtKB protein sequences	UniProtKB, SwissProt, SwissProt Isoforms, TrEMBL, UniProtKB Taxonomic Subsets (13 subgroups, including: bacteria, archaea, eukaryota, SARS-CoV-2, etc.),<br>Reference Proteomes, Representative Proteomes (15, 35, 55, 75), UniProt Reference (UniRef 50, 90 and 100), UniParc, UniProtKB-PDB, MGnify
Patent protein sequences	EPO, JPO, KIPO, UPSPTO
Structures of protein sequences	PDBe, AlphaFold DB
Protein families	Pfam, TIGRFAM, Superfamily, Gene3D, PIRSF, TreeFam, Pfam SARS-CoV-2
Other protein sequences	Enzyme Portal, IntAct, IPD-IMGT/HLA, IPD-KIR, IPD-MHC, MEROPS (MP, MPEP and MPRO), ChEMBL, Quest for Orthologs
ENA nucleotide sequences	ENA sequences for Coding, Non-coding, Barcode, Geospatial, Ribosomal RNA and others (10 subgroups, including: Expressed Sequence Tag,<br>Genome Survey Sequence, etc.)
Ensembl Genomes sequences	Genomes from Bacteria, Fungi, Plants, Metazoa, Protists, WormBase Parasite, SARS-CoV-2
Structures of nucleotide sequences	PDBe
Other nucleotide sequences	IMGT/LIGM-DB, IMGT/HLA (CDS and genomic), IPD-KIR (CDS and genomic), IPD-NHKIR (CDS and genomic), IPD-MHC (CDS and genomic), UniVec
Additional entries available via Dbfetch	EMDB, PDBe-KB, MEDLINE, NCBI Taxonomy, EDAM ontology, HGNC
